# Chosen Vascular Risk Markers in Pseudoexfoliation Syndrome: An Age-Related Disorder

**DOI:** 10.1155/2017/5231095

**Published:** 2017-10-31

**Authors:** Hanna Lesiewska, Agnieszka Łukaszewska-Smyk, Grażyna Odrowąż-Sypniewska, Magdalena Krintus, Aneta Mańkowska-Cyl, Grażyna Malukiewicz

**Affiliations:** ^1^Department of Ophthalmology, The Nicolaus Copernicus University, Ludwik Rydygier's Collegium Medicum, Bydgoszcz, Poland; ^2^Department of Laboratory Medicine, The Nicolaus Copernicus University, Ludwik Rydygier's Collegium Medicum, Bydgoszcz, Poland

## Abstract

**Purpose:**

To evaluate lipids and C-reactive protein serum levels in patients with pseudoexfoliation syndrome (PEX) in the Polish population.

**Methods:**

96 patients were studied with PEX and 79 control subjects. Total cholesterol, triglycerides, high-density lipoprotein (HDL)-cholesterol, low-density lipoprotein (LDL)-cholesterol, non-HDL-cholesterol and CRP serum levels, and TG/HDL-C and TC/HDL-C indexes were assessed.

**Results:**

There were no significant differences in concentration of lipids and values of TC/HDL-C, TG/HDL-C, and non-HDL-C between PEX and control groups. High-sensitivity C-reactive protein was not increased in patients with PEX.

**Conclusions:**

Our results cast doubt on the opinion on the possible PEX and vascular diseases relation. Further studies on this subject are mandatory.

## 1. Introduction

Pseudoexfoliation syndrome (PEX) is an age-related complex systemic disorder of the extracellular matrix affecting the eye and visceral organs. The average worldwide prevalence of PEX is 10%–20% of the general population over the age of 60 years [1, 2]. A population-based study in the Northeastern USA found prevalence of 0.67% in people aged 52–64 years, 2.6% in people aged 65–74 years, and 5% in people aged 78–85 years. In a survey conducted in Iceland, the prevalence of PEX increased from 2.5% in the patients aged 50–59 years to 40.6% in those aged > 80 years [3].

Originally, PEX was thought to be limited to the anterior segment of the eye; some studies have indicated, however, that pseudoexfoliative material may be present in blood vessels and impaired endothelial function can be observed [1, 2]. Since endothelial dysfunction is an independent predictor of future cardiovascular and cerebrovascular events, it might suggest an increased vascular risk in PEX patients [4–6]. Dyslipidemia is a well-established risk factor for cardiovascular and cerebrovascular diseases. Many studies have shown a strong correlation between serum lipids levels and risk of developing vascular events [6–8]. It was found that C-reactive protein (CRP) is significantly associated with cardiovascular disease [9].

The relationship between PEX and vascular diseases has been investigated in many studies [10–16]. Some authors have observed a connection between PEX and cardiovascular or cerebrovascular diseases [4, 10–12, 14, 15]. However, there are also studies reporting that PEX is not associated with arterial hypertension, ischemic heart disease, and cerebrovascular diseases [2, 17–21]. This discrepancy in opinions gave us a spur to assess the relation between PEX syndrome and chosen vascular risk markers in the Polish population.

## 2. Material and Methods

The subjects for this study were recruited from patients who presented to the Department of Ophthalmology, Collegium Medicum, UMK in Bydgoszcz, Poland for cataract surgery. Exclusion criteria were any other ocular diseases except PEX and cataract. Pseudoexfoliation changes were identified by slit lamp examination after pupil dilation as the presence of typical PEX material on the anterior lens surface, iris, or corneal endothelium in either eye. The individuals without any evidence of pseudoexfoliation deposits were taken as the control group. This study has been approved by the local bioethical committee. All patients gave their informed consent for this study.

We followed the methods described in our previous study [22]. Blood samples were collected in all patients after an overnight fast. Plasma was obtained within less than 1 hour to avoid proteolysis and stored deep-frozen (−80°C) in small aliquots until assayed but no longer than 8 months. Total cholesterol (TC), high-density lipoprotein (HDL)-cholesterol, and triglyceride (TG) levels were measured by an autoanalyzer (ARCHITECT ci8200, Abbott Diagnostics, Wiesbaden, Germany) using enzymatic methods. Low-density lipoprotein (LDL)-cholesterol levels were calculated using the Friedewald formula. Moreover, TG/HDL-C, TC/HDL-C, and non-HDL-C as atherogenic indexes were calculated. High-sensitivity CRP (hs-CRP) level was measured using the BN II System nephelometer (High-Sensitivity CRP; Siemens Healthcare Diagnostics, Deerfield, IL, USA), providing excellent precision with the coefficient of variation (CV) reported by the manufacturer of less than 10%. CVs for hs-CRP estimated in our laboratory were below 3.5% and below 4.5% for hs-CRP concentrations below 1 mg/L and above 3 mg/L, respectively. The lower limit of hs-CRP detection was 0.175 mg/L. We divided the patients with PEX into three groups: group A with low risk (hs-CRP concentration less than 1.0 mg/L), group B with average risk (hs-CRP concentration 1.0 to 3.0 mg/L), and group C with high risk of cardiovascular disease (hs-CRP concentration above 3.0 mg/L) according to the American Heart Association and U.S. Centers for Disease Control and Prevention.

The statistical analysis of results was performed using the Kolmogorov-Smirnov test to assess normality of distribution of investigated parameters. Data were expressed as medians with the interquartile range (25th–75th percentiles) according to the distributions of the continuous variables. Differences between the PEX and control groups were analyzed by the Mann–Whitney *U* test for independent samples of nonparametric data. Comparisons of median values between groups were done by ANOVA. The significance level for all statistical tests was 0.05. Statistical analysis was performed using Statistica software (version 8).

## 3. Results

We studied 96 patients with PEX (26 males and 70 females), median age 76 years (Q_1_ = 72; Q_3_ = 82), and 79 age- and sex-matched controls (28 males and 51 females), median age 75 years (Q_1_ = 70; Q_3_ = 80). The concentrations of serum lipids, hs-CRP, and values of atherogenic indexes constituting one of the major cardiovascular risk factors are shown in Table 1. There were no significant differences in concentration of lipids between PEX and control groups. Moreover, we did not observe significant differences for other calculated parameters like TC/HDL-C, TG/HDL-C, and non-HDL-C. High-sensitivity C-reactive protein level was not increased in patients with PEX and was found to be similar to that of controls.

The HDL-C concentration in the PEX female subgroup was statistically higher than in males: 58 mg/dL (Q_1_:48; Q_3_:67) versus 52 mg/dL (Q_1_:40; Q_3_:58), *p* = 0.02. In the control group, we did not find such correlation. The concentrations of other serum lipids, hs-CRP, and values of atherogenic indexes did not differ between males and females, both in PEX and control groups.

We did not observe any significant differences for lipids and atherogenic indexes between the following subgroups: group A with low risk, group B with average risk, and group C with high risk of cardiovascular disease (Table 2).

## 4. Discussion

There are some studies on the possible association between PEX and vascular diseases although their results are inconclusive [10–16]. The risk for cardiovascular and cerebrovascular diseases might be ascribed to the accumulation of pseudoexfoliative fibrils in the arterial wall [2].

It has been proven that one of the several tests in a vascular risk profile, along with tests for cholesterol and triglycerides, is high-sensitivity CRP [6–9].

Studies, that analyzed serum lipids levels and PEX association, provided conflicting results. All these studies are difficult to compare as they vary in size and design. Türkyılmaz et al. found that the mean total cholesterol and triglycerides levels were significantly higher, and mean serum HDL level was lower in the PEX group of 40 patients compared to the controls [23]. We were unable to show an association between the serum lipid levels and PEX syndrome. Our findings are in accordance with the results of Atalar et al. (23 PEX patients) and Spečkauskas et al. (152 PEX patients), who did not reveal the differences in lipid levels in PEX patients [2, 24]. The latter authors found no clear PEX association with triglyceride and HDL cholesterol levels as well as ischemic heart disease after controlling for effect of age in the population-based study. Presence of PEX was not significantly associated with the blood concentration of high-density lipoproteins and cholesterol in the study of Jonas et al. comprising 69 PEX patients [25].

There is also discrepancy regarding CRP levels in PEX. Elevated plasma hs-CRP levels have been reported in patients with PEX by Sorkhabi et al. [26]. Nevertheless, the study of Kymionis et al. as well as Yüksel et al. revealed that serum CRP levels were not elevated in patients with PEX [27, 28]. This is in accordance with our results which showed that serum CRP levels were not increased in patients with PEX and were found to be similar to that of controls. The conflicting results of these studies may be due to the selection bias. Similarly to Sorkhabi et al., in our study, we strictly excluded all conditions which may affect the levels of inflammatory biomarkers [26].

Our research casts doubt on the possibility of PEX and cardiovascular and cerebrovascular disease association. These findings suggest that patients with this syndrome do not suffer from increased comorbidity and mortality.

## 5. Conclusions

Serum lipids and hs-CRP levels nor values of atherogenic index hs-CRP were elevated in PEX patients in the Polish population. These findings raise doubt on the opinion on the possible PEX and vascular disease relation. As ours and other studies results are inconclusive, the causes and connections of PEX still remain unexplained and further studies on this subject are required.

## Figures and Tables

**Table 1:** Biochemical parameters in PEX patients and controls.

Parametres	PEX patients' median (Q_1_–Q_3_) *n* = 96	Control median (Q_1_–Q_3_) *n* = 79	*p*
TC (mg/dL)	199 (176–240)	213 (178–245)	0.25
HDL-C (mg/dL)	55 (45–66)	53 (46–64)	0.39
TG (mg/dL)	125.5 (83.5–161.5)	114 (88–152)	0.61
LDL-C (mg/dL)	114.5 (93.5–152)	128 (103–156)	0.14
Non-HDL-C (mg/dL)	139 (115.5–181)	158 (128–193)	0.13
TC/HDL-C	3.73 (2.95–4.50)	3.78 (3.28–4.54)	0.26
TG/HDL-C	2.2 (1.39–3.30)	2.07 (1.45–3.45)	0.83
hs-CRP mg/l	1.53 (0.75–3.71)	1.57 (0.74–3.44)	0.90

**Table 2:** Lipid concentration and atherogenic indexes in relation to hs-CRP levels in patients with PEX.

	Group A median (Q_1_–Q_3_) *n* = 34	Group B median (Q1–Q3) *n* = 35	Group C median (Q1–Q3) *n*=27	*p*
TC (mg/dL)	200 (182–245)	190 (164–217)	204 (161–244)	0.18
TG (mg/dL)	114 (83–181)	115 (81–148)	130 (114–156)	0.95
HDL-C (mg/dL)	59 (48–68)	54 (45–66)	51 (44–58)	0.35
LDL-C (mg/dL)	115 (95–152)	104 (92–142)	130 (98–161)	0.24
non-HDL-C (mg/dL)	154 (127–190)	126 (115–172)	157 (118–191)	0.26
TC/HDL-C	3.9 (3.4–4.5)	3.3 (2.7–4.8)	4.1 (3.2–4.8)	0.63
TG/HDL-C	2.1 (1.4–3.4)	2.2 (1.5–3.1)	2.6 (1.7–3.5)	0.70
